# Socioeconomic patterning of vaping by smoking status among UK adults and youth

**DOI:** 10.1186/s12889-020-8270-3

**Published:** 2020-02-10

**Authors:** Michael J. Green, Linsay Gray, Helen Sweeting, Michaela Benzeval

**Affiliations:** 1 MRC/CSO Social & Public Health Sciences Unit, University of Glasgow, 200 Renfield Street, Glasgow, G2 3AX UK; 2 Institute for Social and Economic Research, University of Essex, Colchester, CO3 3LG UK

**Keywords:** Adults, E-cigarettes, Smoking, Socioeconomic position, Vaping, Youth

## Abstract

**Background:**

Smoking contributes significantly to socioeconomic health inequalities. Vaping has captured much interest as a less harmful alternative to smoking, but may be harmful relative to non-smoking. Examining inequalities in vaping by smoking status, may offer insights into potential impacts of vaping on socioeconomic inequalities in health.

**Methods:**

Data were from 3291 youth (aged 10–15) and 35,367 adults (aged 16+) from wave 7 (2015–17) of the UK Household Longitudinal Study. In order to adjust for biases that could be introduced by stratifying on smoking status, marginal structural models were used to estimate controlled direct effects of an index of socioeconomic disadvantage (incorporating household education, occupation and income) on vaping by smoking status (among adults and youth), adjusting for relevant confounders and for selection into smoking states. We also estimated controlled direct effects of socioeconomic disadvantage on being an ex-smoker by vaping status (among adult ever-smokers; *n* = 18,128).

**Results:**

Socioeconomic disadvantage was associated with vaping among never smoking youth (OR for a unit increase in the socioeconomic index: 1.17; 95%: 1.03–1.34), and among ex-smoking adults (OR: 1.17; 95% CI: 1.09–1.26), with little to no association among never smoking (OR: 0.98; 95% CI: 0.91–1.07) and current smoking (OR: 1.00; 95% CI: 0.93–1.07) adults. Socioeconomic disadvantage was also associated with reduced odds of being an ex-smoker among adult ever-smokers, but this association was moderately weaker among those who vaped (OR: 0.88; 95% CI: 0.82–0.95) than those who did not (OR: 0.82; 95% CI: 0.80–0.84; *p*-value for difference = 0.081).

**Conclusions:**

Inequalities in vaping among never smoking youth and adult ex-smokers, suggest potential to widen health inequalities, while weaker inequalities in smoking cessation among adult vapers indicate e-cigarettes could help narrow inequalities. Further research is needed to understand the balance of these opposing potential impacts, and how any benefits can be maximised whilst protecting the vulnerable.

## Background

Use of electronic cigarettes (e-cigarettes) rose precipitously in the UK from 2011 [1], before recently plateauing with approximately 6% of adults currently using them [2]. While health consequences of long-term e-cigarette use (or vaping) are largely unknown [1–4], expert opinion regards vaping as substantially less harmful than smoking [1, 5]. Mounting evidence supports this view [2, 6–8] and that vaping may aid smoking cessation [2, 9–11]. Nevertheless, concerns remain [3, 12], particularly around risks of vaping introducing youth to nicotine and cigarette use [13–16], though so far regular (at least weekly) vaping by youth in the UK has been rare [17, 18].

The implications of e-cigarettes for socioeconomic inequalities in smoking and hence health have received limited attention [2]. A recent review found socioeconomic inequalities in awareness and use (ever or current) of e-cigarettes, but findings were very mixed, with some studies, especially those rated as higher quality, suggesting greater awareness and use in higher income, better educated groups, but others finding the reverse, or no clear differences [19]. One possible reason for inconsistent findings, may be varying consideration of smoking status, with some studies focusing on the general population [20–22], others stratifying by smoking status [20, 23], and others focusing only on current or former smokers [24–26].

Stratifying by smoking status is critical to understanding potential impacts of e-cigarettes on health inequalities as conclusions will depend on how vaping interacts with smoking behaviour. Table 1 identifies six groups based on e-cigarette and cigarette use and which we use for analysis in this paper. Vaping among never-smokers represents a potential public health concern: ‘safer than smoking’ may not be the same thing as ‘safe’ [2, 3, 27, 28]. Vaping among youth who have never smoked may be especially concerning as nicotine addiction established at this life-stage could be long-lasting, potentially increasing the likelihood of cigarette use over the life-course and/or risk for psychiatric disorders, future substance use and poor later life cognition [2, 29–31]. If vaping is more likely among disadvantaged never smokers it could indicate potential widening of health inequalities (unless vaping replaces and does not lead to take-up of smoking).

**Table 1:** Cross Classifications of E-Cigarette and Cigarette Use

		Cigarette Use^a^		
		Never Smoker	Ex-Smoker	Current Smoker
E-Cigarette Use	Non-Vaper^b^	Non-Users	Ex-Smoker who does not vape	Smoker who does not vape
	Vaper	Vaper who has never smoked	Ex-Smoker who vapes	Dual-Use

^a^For youth, considering low prevalence, ex-smokers and current smokers were combined into a single category of ever-smokers

^b^Non-vaping includes ex and never vapers as we did not think it important to distinguish these categories for our purposes here

Perhaps most importantly, management of nicotine addiction with e-cigarettes only (i.e. vaping among ex-smokers) is likely to be substantially less harmful than with smoking [1, 2, 6, 7], and vaping by ex-smokers may often represent intentional replacement of cigarettes with e-cigarettes for health reasons [32]. There could also be other reasons for switching such as relative price, preference, or differences in regulation as to where the behaviour is allowed. Vaping ex-smokers could also include long-time ex-smokers who have returned to nicotine with e-cigarettes, perhaps instead of relapse to smoking.

Historically, more advantaged individuals have been more successful in smoking cessation than those who are more disadvantaged [33], but this inequality has reduced in recent years, and e-cigarettes have been suggested by others as one possible explanation for this trend [34]. Thus, while we might expect to see inequalities in whether smokers have quit, a key question is whether inequalities in ex-smoking status are smaller with vaping than without [19]. Such a pattern would indicate a potential for e-cigarettes to narrow inequalities in smoking (and therefore health). However, vaping instead of smoking could still carry some residual health risk [35] relative to using neither, so socioeconomic patterning of vaping among ex-smokers, may still represent some residual potential to widen health inequalities.

Vaping among current smokers probably often indicates some interest in quitting and/or concern over the health risks associated with smoking (perhaps less so among youth where it may just indicate a general propensity for experimental or risky behaviour). Although dual-use of e-cigarettes and cigarettes may be motivated by harm reduction, there is little evidence that dual-use is any healthier than smoking only (since dual-use still includes smoking, even if it reduces the number of cigarettes smoked) [7]. Dual-users could represent a prime-target group who may be amenable to interventions to aid smoking cessation (e.g. by switching solely to vaping, or using vaping as a step towards complete cessation of nicotine use). Understanding the social patterning of vaping among current smokers could help understand the potential impacts of dual-use targeted interventions on inequalities in smoking.

**Fig. 1:**
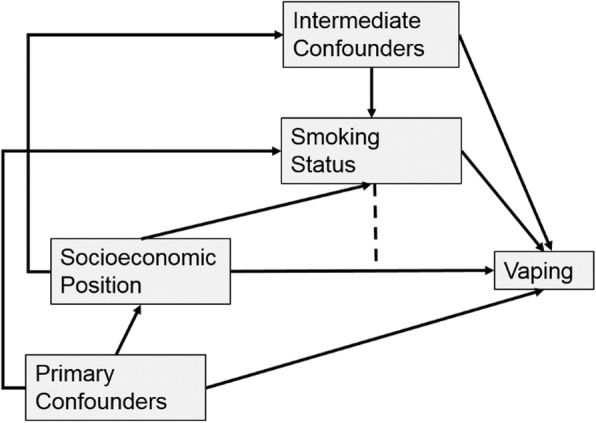
Causal Diagram for analyses of SEP, smoking and vaping

Nevertheless, while stratification by smoking status is important for interpretations of inequalities in vaping, stratification can also introduce a phenomenon known as collider bias [36, 37], which can arise when conditioning on a variable that is determined by both the exposure of interest and other variables that determine the outcome of interest. This is illustrated in Fig. 1, showing socioeconomic position (SEP) as a determinant of vaping, with potential (primary) confounders and smoking as a mediator, i.e. it is determined by SEP, and, in turn, may determine vaping. The dashed line indicates that the effect of SEP on vaping may differ by smoking status. Collider bias can arise if there are confounders of the effect of smoking on vaping (denoted intermediate confounders in the diagram), such as individual smoking histories, or parental smoking/vaping (for youth). Since smoking is determined by both SEP and the intermediate confounders, stratifying (or otherwise conditioning) on it induces a spurious association between SEP and the intermediate confounders and can bias estimates of association between SEP and vaping. If there were no causal relationship between SEP and the intermediate confounders then adjusting for these in stratified analyses would be sufficient. However, if any of the intermediate confounders are also determined by SEP as shown in Fig. 1 (they need not all be determined by SEP), then adjusting for them in stratified analyses will remove part of the effect of interest, while stratifying without adjustment will induce collider bias, and estimates will be biased either way [38, 39]. In these circumstances, marginal structural models can be used to estimate controlled direct effects (CDEs) [38, 39] within strata of smoking (assuming no unmeasured confounding).

Thus, our paper, based on a representative UK survey, uses marginal structural models to estimate socioeconomic inequalities in vaping and smoking cessation, specifically addressing the three research questions presented in Table 2.

**Table 2:** Research questions and the samples and variables analysed

	RQ1: Is vaping associated with SEP among youth and does this vary by smoking status?	RQ2: Is vaping associated with SEP among adults and does this vary by smoking status?	RQ3: Are socioeconomic inequalities in ex-smoking smaller for ever-smoking adults who vape than those who do not?
Sample	Youth aged 10–15	Adults aged 16+	Adults aged 16+
Exposure	SEP	SEP	SEP
Outcome	Vaping (overall and by smoking status)	Vaping (overall and by smoking status)	Ex-Smoking (among ever-smokers who do and do not vape)
Primary Confounders	Country Ethnicity Family Structure Interview Date	Gender Age Country Ethnicity Lives with Spouse/Partner Children in Household Interview Date	Gender Age Country Ethnicity Lives with Spouse/Partner Children in Household Interview Date
Intermediate Confounders	Gender^a^ Age^a^ Parental Smoking Parental Vaping	Current smoker at 1, 2 & 5 years previous	Current smoker at 1, 2 & 5 years previous

^a^These variables were probably not caused by SEP as indicated in Fig. 1, but for youth these are also unlikely to be common causes of SEP and vaping, so were included here, rather than as primary confounders

## Methods

### Sample

**Fig. 2:**
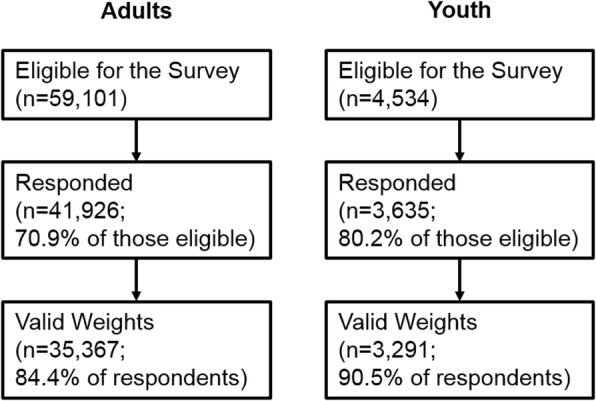
Flowcharts showing sample response rates

Respondents were from the 7th Wave of Understanding Society: the UK Household Longitudinal Study, a household panel survey which performs annual interviews on residents of UK households [40]. The survey is based on a stratified, clustered, equal probability sample of UK residential addresses with boost samples for minority ethnic groups added at waves 1 and 6 (the Northern Ireland sample was not clustered). Fieldwork was conducted between January 2015 and May 2017. The flowchart in Fig. 2 shows how the analytical samples were arrived at: 41,926 adult individuals (aged 16+) responded (70.9% of those eligible). Youth aged 10–15 years in responding households were also eligible and 3635 completed questionnaires (80.2% of those eligible). All analyses were conducted in Mplus 8 [41] and applied weighting to adjust for survey design and non-response, to be representative of the UK population (though this excluded 6559 adults and 344 youth without valid survey weights) [42]. Multiple imputation was applied to include all respondents with valid weights [43], and 93.2% of the adults and 72.1% of the youth with valid weights had complete data on all analysis variables. Multiple imputation was conducted with an unconstrained model of all analysis variables (i.e. allowing each variable to predict all others) and results were averaged across 25 imputed datasets using Rubin’s rules [41].

### Measures

Vaping for adults and youth was classified by yes or no responses to the question: “Do you ever use electronic cigarettes (e-cigarettes)?” which is interpreted as representing current vaping, though this could include anything from regular to infrequent vaping. With regard to traditional cigarettes, respondents self-reported as either current, ex or never smokers. Due to low prevalence of smoking, youth were coded as either never or ever smokers.

SEP was represented by an index constructed from three measures: educational level, occupational status and net income, based on household measures. Highest educational level was coded: degree or higher; A-Level or equivalent; General Certificate of Secondary Education (GCSE) or equivalent; or no qualifications. Occupational status was coded using the UK National Statistics Socioeconomic Classification (NS-SEC) as: managerial or professional; intermediate; routine; or not employed. The most advantaged occupation/educational level was used from couple households. Net household income was equivalised for household composition and split into quartiles. Each of these three variables was coded from 0 to 3 (most advantaged-least advantaged) and then summed into an index ranging from 0 to 9 (with higher scores representing greater disadvantage).

Potential confounding variables were measured as follows. Gender was binary coded as male or female. Age was grouped into five categories for adults (16–24, 25–34, 35–54, 55–74, 75+ years), and categorised by single year for youth. Ethnicity was self-reported and binary coded (White UK vs ethnic minority). Adults were coded as living with a spouse or partner vs single, and living with or without children (aged 0–15 years) in the household. For youth, family structure was coded as having either couple or single parents. We included an indicator of UK country (England, Wales, Scotland, Northern Ireland) and a continuous measure of interview date (as there were slight temporal trends in vaping within the period of fieldwork). For youth analysis, parental smoking and vaping were coded according to the greatest level of use from either parent. For adult analyses we included variables indicating whether they were current smokers in earlier waves of the survey (1, 2 and 5 years previous, as this was when smoking status had been ascertained).

### Statistical analyses

Initially, for each question we report the unadjusted, observed association between each exposure and outcome of interest. We then employ marginal structural models to give estimates of effect that are adjusted for observed confounding. This procedure employs propensity weights aiming to balance confounders across exposure levels but only to the extent that any imbalance in confounders is not caused by the exposure. Thus, we start with preparatory models predicting the exposure of interest (SEP) and the intermediate exposure (smoking status) and use predicted probabilities from these to create analysis weights that are employed to adjust our estimates of the effects of the exposures on the outcomes. Table 2 specifies how the analysis samples and variables are used for each research question. For each question we began with estimating two preparatory ordinal logistic regression models predicting SEP (the exposure), one with and one without adjustment for the primary confounders (as specified in Table 2). An exposure weight was calculated by dividing the predicted probability of each individual’s observed SEP value from the model without confounders by that from the model with confounders.

For research question 1 about youth vaping, further preparatory logistic regression models were used to predict whether youth had ever smoked, one based only on SEP, and another based on SEP and both the primary and the additional intermediate confounders. Gender and age were not considered likely determinants of household SEP for youth and so were included as intermediate confounders only. An intermediate exposure weight was calculated by dividing the predicted probability of an individual’s observed smoking status from the model based only on SEP by that from the model with all confounders. This intermediate exposure weight was multiplied together with the first exposure weight (and the survey design/non-response weights) to create a final analysis weight, designed to remove observed confounding but avoid collider bias. The estimates of interest, CDEs of SEP on vaping, were then obtained via a logistic regression including only SEP, ever smoking and their interaction, weighted by the analysis weight. For presentation, coefficients for SEP and its interaction with smoking status have been combined to obtain estimates for SEP within smoking strata. Thus, in the final model, the analysis weights balance differences in confounders within smoking strata that are not caused by SEP.

For research question 2 a similar approach was used but required an additional step, to obtain an extra intermediate weight, because previous smoking would only occur among ever smokers and zero probability of being never smokers is problematic for weighting. Following calculation of the exposure weights, we estimated two logistic regression models predicting ever smoking, one based only on SEP, and the other based on SEP and all the primary confounders. The first intermediate weight was calculated by dividing the predicted probability of the respondents’ actual smoking level (ever vs never) from the first model by that from the second model. We then estimated two further logistic regression models predicting current smoking among ever-smokers, one based only on SEP and the second based on SEP and both primary and intermediate confounders. The second intermediate weight was calculated for ever smokers by dividing the predicted probability of their observed smoking status from the first of these models by that from the second. For never smokers this second intermediate weight was set to 1. The analysis weight was calculated by multiplying the exposure weight together with both intermediate weights (and the survey design/non-response weights). CDEs of SEP on vaping were estimated with a weighted logistic regression (using the analysis weight) of vaping on SEP, smoking status and their interaction. Again, for presentation, coefficients for SEP and its interaction with smoking status have been combined to obtain estimates for SEP within smoking strata. As above, the weights balance differences in confounders within smoking strata that are not caused by SEP.

For research question 3, the above process was repeated except that the second intermediate weight was calculated from models predicting vaping status among ever smokers, and the final analysis was a weighted logistic regression of non-smoking status on SEP, vaping, and their interaction among ever smokers only (since all never smokers are non-smokers). Thus, the weights are intended to balance for differences in confounders between ever smokers who vape and those who do not that are not caused by SEP.

Odds ratios (OR) for SEP represent the additional risk per unit increase in the index of socioeconomic disadvantage (i.e. the average difference between one socioeconomic category and the next most advantaged). Standard errors from the weighted logistic regression models were adjusted for clustering of respondents within households in order to calculate appropriate 95% confidence intervals for the effects of SEP. We performed sensitivity analyses with weights truncated at their 95th percentile to check whether results were dependent on extreme weights in a small number of cases.

## Results

Table 3 shows sociodemographic patterning of vaping among youth aged 10–15 by whether or not they have ever smoked. Overall, vaping was rare among never smokers but more common among ever smokers (1.4% compared to 28.4%). Youth vaping was more common among males than females, among older youth, those with single parents, and those whose parents used e-cigarettes, were in disadvantaged occupation categories, or had lower incomes. Aside from the patterning by gender and parental vaping, all these associations were diluted but held among never smokers. Among the small group of ever smokers (*n* = 247) only patterning by gender and age was still evident.

**Table 3:** Sociodemographic patterning of vaping among youth (aged 10–15) by smoking status^a^

	Sociodemographic Characteristics		Vaping Among Never Smokers			Vaping Among Ever Smokers			All Vaping		
	N	%	Yes N	%	*P*-Value	Yes N	%	*P*-Value	Yes N	%	*P*-Value
Total	3291	100	42	1.4	–	70	28.4	–	112	3.4	–
No Vaping	3179	96.6									
Vaping	112	3.4									
Never Smokers	3044	92.5							42	1.4	< 0.001
Ever Smokers	247	7.5							70	28.4	
Male	1629	49.5	25	1.7	0.146	43	34.7	0.032	68	4.2	0.018
Female	1662	50.5	16	1.1		27	22.2		44	2.7	
Age 10	520	15.8	4	0.8	< 0.001	0	1.6	0.069	4	0.8	< 0.001
Age 11	596	18.1	0	0.0		0	0.2		0	0.1	
Age 12	561	17.0	4	0.7		0	0.4		4	0.7	
Age 13	493	15.0	5	1.1		8	28.3		13	2.7	
Age 14	588	17.9	12	2.5		28	32.8		41	6.9	
Age 15	533	16.2	15	3.6		34	30.4		49	9.2	
England	2831	86.0	38	1.4	0.838	59	27.8	0.412	97	3.4	0.607
Wales	111	3.4	2	1.9		2	24.7		4	2.8	
Scotland	263	8.0	2	0.8		5	26.1		7	3.0	
Northern Ireland	86	2.6	1	1.3		4	56.3		5	5.9	
White UK	2704	82.2	33	1.3	0.437	62	28.7	0.668	96	3.5	0.420
Ethnic Minority	587	17.8	8	1.5		8	27.5		17	2.8	
Couple Parents	2474	75.2	20	0.9	< 0.001	43	29.4	0.686	63	2.6	< 0.001
Single Parent	817	24.8	21	3.0		27	27.0		49	6.0	
Parents Never Smokers	1175	35.7	13	1.1	0.193	23	33.3	0.505	35	3.0	0.126
Ex-Smoking Parent	1301	39.5	14	1.2		26	25.1		40	3.1	
Current Smoking Parent	815	24.8	15	2.1		22	28.5		37	4.5	
No Parental Vaping	2903	88.2	36	1.3	0.711	55	27.5	0.495	91	3.1	0.021
Parental Vaping	388	11.8	6	1.6		15	32.5		21	5.5	
Degree	1739	52.8	17	1.1	0.445	34	30.4	0.737	51	2.9	0.331
A-Level or equivalent	676	20.5	11	1.8		13	25.6		24	3.6	
GCSE or equivalent	782	23.8	12	1.7		22	29.3		34	4.4	
No Qualifications	94	2.9	1	1.2		2	17.7		3	3.3	
Managerial/Professional	1347	40.9	4	0.3	< 0.001	21	24.9	0.275	25	1.8	< 0.001
Intermediate	489	14.9	9	1.9		13	35.7		21	4.4	
Routine	539	16.4	14	2.8		17	38.9		31	5.7	
Not employed	916	27.8	15	1.8		20	23.4		35	3.8	
Highest Income Quartile	473	14.4	1	0.3	0.002	7	26.1	0.586	9	1.9	< 0.001
2nd Quartile	850	25.8	4	0.5		13	22.2		17	2.1	
3rd Quartile	1094	33.2	17	1.6		21	31.2		37	3.5	
Lowest Income Quartile	875	26.6	19	2.4		29	30.9		48	5.5	

^a^Data weighted for attrition and over-sampling, and results averaged across 25 imputed datasets

Table 4 shows the sociodemographic patterning of vaping among adults (aged 16+) by smoking status. Vaping by never smokers was very rare at 1.1%, more common among ex-smokers (6.9%) and markedly more common among current smokers (27.1%). Vaping was more common overall among males, at younger ages (especially ages 25–54), among White UK respondents, for those who were single, those who had children in the household, those in disadvantaged groups (though the most disadvantaged categories for education and occupation had relatively low rates of vaping -perhaps due to conflation with age) and those who had smoked in previous waves. However, most of these patterns varied by smoking status, for example, the association with education was reversed among current smokers.

**Table 4:** Sociodemographic patterning of vaping among adults (aged 16+) by smoking status^a^

	Sociodemographic Characteristics		Vaping Among Never Smokers			Vaping Among Ex-Smokers			Vaping Among Current Smokers			All Vaping		
	N	%	Yes N	%	*P*-Value	Yes N	%	*P*-Value	Yes N	%	*P*-Value	Yes N	%	*P*-Value
Total	35,367	100.0	189	1.1	–	839	6.9	–	1597	27.1	–	2624	7.4	–
No Vaping	32,743	92.6												
Vaping	2624	7.4												
Never Smokers	17,238	48.7										189	1.1	< 0.001
Ex-Smokers	12,238	34.6										839	6.9	
Current Smokers	5891	16.7										1597	27.1	
Male	16,930	47.9	115	1.5	< 0.001	452	7.2	0.092	799	27.0	0.907	1366	8.1	< 0.001
Female	18,437	52.1	74	0.8		387	6.5		798	27.2		1258	6.8	
16–24	4758	13.5	95	2.8	< 0.001	75	15.3	< 0.001	205	25.3	< 0.001	375	7.9	< 0.001
25–34	4801	13.6	32	1.3		156	12.5		275	24.3		463	9.6	
35–54	11,696	33.1	36	0.6		387	9.7		640	28.8		1063	9.1	
55–74	10,415	29.4	20	0.5		196	4.3		452	29.6		668	6.4	
75+	3697	10.5	5	0.3		24	1.3		25	12.1		54	1.5	
England	29,815	84.3	172	1.2	0.063	711	6.8	0.443	1311	27.1	0.322	2194	7.4	0.094
Wales	1681	4.8	7	0.9		42	7.9		90	25.9		139	8.2	
Scotland	2911	8.2	6	0.4		72	7.4		157	29.3		235	8.1	
Northern Ireland	960	2.7	4	0.8		15	5.2		39	22.5		58	6.0	
White UK	30,781	87.0	151	1.1	0.322	772	6.9	0.766	1452	27.7	0.004	2376	7.7	< 0.001
Ethnic Minority	4586	13.0	38	1.3		66	6.8		144	22.4		248	5.4	
Has Spouse/Partner	21,222	60.0	71	0.7	< 0.001	548	6.5	0.035	831	28.7	0.007	1450	6.8	< 0.001
Single	14,145	40.0	118	1.6		291	7.6		765	25.6		1174	8.3	
No Children in Household	24,919	70.5	125	1.1	0.442	551	6.1	< 0.001	1095	27.7	0.179	1771	7.1	0.001
Children in Household	10,448	29.5	64	1.2		288	9.1		502	26.0		853	8.2	
Degree	16,031	45.3	78	0.9	0.121	388	6.8	< 0.001	471	29.1	0.026	937	5.8	< 0.001
A-Level or equivalent	8012	22.7	48	1.2		212	8.5		421	26.9		680	8.5	
GSCE or equivalent	8389	23.7	49	1.4		196	6.9		556	27.1		801	9.5	
No Qualifications	2935	8.3	14	1.3		43	3.6		149	22.8		206	7.0	
Managerial/Professional	11,536	32.6	63	1.0	0.018	307	7.6	< 0.001	368	29.8	< 0.001	739	6.4	< 0.001
Intermediate	4755	13.4	20	0.9		126	7.9		254	30.1		400	8.4	
Routine	6350	18.0	50	1.7		183	10.4		463	27.9		696	11.0	
Not employed	12,726	36.0	56	1.0		223	4.6		512	23.8		790	6.2	
Highest Income Quartile	9429	26.7	59	1.1	0.481	186	5.5	0.002	249	28.4	0.016	495	5.2	< 0.001
2nd Quartile	9427	26.7	51	1.1		257	7.7		433	29.4		741	7.9	
3rd Quartile	9004	25.5	50	1.2		223	7.3		482	27.1		755	8.4	
Lowest Income Quartile	7507	21.2	29	0.9		173	7.1		432	24.5		634	8.4	
Non-Smoker 1 year ago	29,290	82.8		–		550	4.8	< 0.001	164	24.1	0.068	902	3.1	< 0.001
Smoker 1 year ago	6077	17.2				289	33.3		1433	27.5		1722	28.3	
Non-Smoker 2 years ago	28,940	81.8		–		419	3.8	< 0.001	182	24.3	0.081	790	2.7	< 0.001
Smoker 2 years ago	6427	18.2				419	32.6		1414	27.5		1834	28.5	
Non-Smoker 5 years ago	28,036	79.3		–		225	2.2	< 0.001	143	19.3	< 0.001	556	2.0	< 0.001
Smoker 5 years ago	7331	20.7				614	28.2		1454	28.2		2068	28.2	

^a^Data weighted for attrition and over-sampling, and results averaged across 25 imputed datasets

Table 5 shows estimates of socioeconomic inequalities in vaping by smoking status among youth aged 10–15 from both unadjusted and marginal structural models. Unadjusted associations and CDE estimates both indicated vaping was more likely for disadvantaged youth. The associations and effects estimates tended to be weaker among ever smokers than among never smokers, but not significantly so.

**Table 5:** Estimates of SEP effects on vaping by smoking status

	Unadjusted Association between SEP and vaping			CDE estimates of SEP on vaping		
	OR	95% CI	*P*-value for difference from association among never smokers	OR	95% CI	*P*-value for difference from effect among never smokers
*Youth (aged 10–15)*						
Never Smokers	1.18	1.06–1.32	–	1.17	1.03–1.34	–
Ever Smokers	1.08	0.90–1.29	0.380	1.03	0.82–1.29	0.309
All^a^	1.16	1.06–1.28	–	1.14	1.01–1.29	–
*Adults (aged 16+)*						
Never Smokers	1.02	0.95–1.10	–	0.98	0.91–1.07	–
Ex-Smokers	0.97	0.94–1.00	0.193	1.17	1.09–1.26	< 0.001
Current Smokers	0.95	0.92–0.98	0.081	1.00	0.93–1.07	0.781
All^a^	1.05	1.04–1.07	–	1.12	1.06–1.18	–

^a^The unadjusted association for all respondents is not adjusted for smoking status, whereas the CDE estimate is adjusted for smoking status (and other primary and intermediate confounders)

Table 5 also shows socioeconomic inequalities in vaping by smoking status among adults. The unadjusted associations indicated that socioeconomic disadvantage was associated with a greater likelihood of vaping overall, but stratifying by smoking status this association disappeared among never smokers and was reversed among ex and current smokers. After adjusting for smoking and other confounders in the CDE estimates, socioeconomic disadvantage seemed even more strongly linked to vaping. However, this was clearly concentrated among ex-smoking adults, with little to no effects among never or current smoking adults, suggesting that the observed associations were affected by either confounding or collider bias.

Table 6 shows socioeconomic inequalities in ex-smoking among ever-smoking adults by vaping status. Socioeconomic disadvantage was associated with reduced odds of being an ex-smoker and this observed association differed little by vaping status. However, after adjustment for confounders, CDE estimates of SEP on ex-smoking were marginally weaker among those who vaped than those who did not (*p* = 0.081).

**Table 6:** Estimates of SEP effects on ex-smoking by vaping status among ever-smoking adults (aged 16+)

	Unadjusted Association between SEP and ex-smoking status			CDE estimates of SEP on ex-smoking status		
	OR	95% CI	*P*-value for difference from association among non-vapers	OR	95% CI	*P*-value for difference from effect among non-vapers
Non-Vaper	0.87	0.85–0.88	–	0.82	0.80–0.84	–
Vaper	0.86	0.82–0.90	0.752	0.88	0.82–0.95	0.081
All^a^	0.87	0.86–0.89	–	0.83	0.81–0.85	–

^a^The unadjusted association for all ever-smoking respondents is not adjusted for vaping status, whereas the CDE estimate is adjusted for vaping status (and other primary and intermediate confounders)

Findings from sensitivity analyses with truncated weights were largely consistent (and therefore not shown), but the CDE estimate for SEP on vaping among adult ex-smokers (from Table 5) was attenuated (from OR: 1.17; 95% CI: 1.09–1.26 to OR: 1.06; 95% CI: 1.02–1.10) and less clearly differentiated from the estimate of no effect among never-smokers (*p* = 0.173). The overall CDE estimate for SEP on vaping among all adult respondents was also attenuated (from OR: 1.12; 95% CI: 1.06–1.18 to OR: 1.02; 95% CI: 1.00–1.05).

## Discussion

This robust analysis of data from a large and representative UK survey provides evidence of complex variations in vaping inequalities by age and smoking status. Our findings concur with reports of low vaping prevalence among youth, especially youth who have never smoked [1, 2, 17, 18, 32, 44]. Vaping was more likely for youth in disadvantaged than more advantaged groups, especially among youth who had never smoked, though concerns should be tempered by the low overall prevalence [45], and because some of this unequal take-up of vaping could be replacing unequal take-up of smoking, which would be more health damaging. Regarding adults, like others we found that more advantaged ever-smokers seemed more likely to use e-cigarettes, and to have quit smoking [1, 2, 24, 33], but the association between socioeconomic advantage and vaping among current smokers disappeared with adjustment for confounding and collider biases. Our adjusted analyses indicated that socioeconomic disadvantage increased the likelihood of vaping among ex-smokers, while there was little to no effect of SEP on vaping among never or current smokers.

With respect to the association between socioeconomic advantage and ex-smoking among ever-smoking adults, confounding adjustment showed slightly weaker effects of SEP among ever smokers who vaped than those who did not. Although only marginally different, this is encouraging and worthy of further research. Studies already suggest that vaping can aid smoking cessation [2, 9–11], but if e-cigarettes are especially appealing or helpful to disadvantaged smokers, then this could have a welcome long-term impact of reducing health inequalities. There have been reductions in inequalities in successful smoking cessation in recent years [34], and some have attributed this trend to e-cigarettes. Our findings were somewhat consistent with this notion, but did not examine trends in cessation specifically, and further study of how inequalities in smoking cessation are impacted over time by e-cigarettes is needed.

Given the cross-sectional nature of the data, the adult findings (regarding both inequalities in vaping and cessation) could have been brought about by disadvantaged ex-smokers being more likely than advantaged ex-smokers to take up vaping (i.e. relapsing to nicotine but not cigarette use), as well as by disadvantaged smokers switching to vaping. We have not yet been able to investigate longitudinal dynamics between e-cigarette and cigarette use [9, 15, 46]. This will be possible with future waves of the study and clarifying inequalities in transitions between particular smoking and vaping states will be an important issue for such longitudinal research.

Other limitations included the question on vaping not distinguishing between different types of e-cigarette devices, or different frequencies of vaping [9, 47]. Associations might differ in either direction if we were able to further stratify by such variables, and frequency and device types could also affect how vaping should be interpreted in terms of public health impact (e.g. infrequent vaping may be less effective for cessation [4]). Nevertheless, theory regarding vaping leading to smoking does not necessarily rely on frequent use, mere experience of the social performance of nicotine use may be sufficient to facilitate transition to smoking [48]. Moreover, without data on intentions or attempts to quit, our comparisons of ex-smoking against current smoking potentially conflate socioeconomic differences in intentions to quit with socioeconomic differences in successful quitting. Youth was defined as ages 10–15 due to the structure of the survey data, but patterns could be distinct among young adults aged 16+ (who were grouped with adults here), though the age-range covered here is similar to that in other studies of UK youth [17, 18]. Further, while we have adjusted for many relevant confounders (including recent smoking history) while preserving the effects of SEP on these confounders, the effect estimates presented here assume no unmeasured confounding [46]. We did not have information on prior vaping, which could have particularly biased analyses of smoking cessation, as respondents recorded here as ex-smokers who do not vape could have already used vaping as an intermediate step to help them quit nicotine completely [49].

Nevertheless, understanding impacts of vaping on socioeconomic inequalities in smoking is especially important considering that few population-level tobacco control interventions (except taxation) have had much success in this regard [50–52]. The role of e-cigarettes in smoking cessation continue to be a focus of policy and regulatory debate. Interventions to reduce smoking should aim to maximise potential benefits of e-cigarettes and consider possible impacts on inequalities. Taxation that keeps cigarette prices high relative to e-cigarettes may be an avenue worthy of study, and could help encourage disadvantaged smokers to switch to e-cigarettes or use vaping as a step towards no nicotine use. Measures may also be needed to protect vulnerable groups and minimise potential harms, e.g. by restricting availability of e-cigarettes to youth or at least closely monitoring inequalities in uptake among youth [2].

## Conclusions

Assuming smoking is more harmful than vaping and that vaping is more harmful than no use of nicotine, the socioeconomic inequalities that we found in vaping among never smoking youth and ex-smoking adults could potentially lead to some future widening of socioeconomic inequalities in health. Conversely, we found weaker inequalities in smoking cessation among smokers who vaped, and this could have an opposing effect leading to narrowing of health inequalities. In other research, the potential impacts of e-cigarettes on smoking prevalence and health have been estimated using various predictive models with different assumptions [3, 53–55], and similar efforts to estimate their likely impact on inequalities in smoking and health would now seem advisable to understand the potential net impacts of these opposing trends.

## Data Availability

The datasets analysed during the current study are available from the UK Data Service repository: https://beta.ukdataservice.ac.uk/datacatalogue/studies/study?id=6614 DOI: 10.5255/UKDA-SN-6614-12.

## Abbreviations

CDE: Controlled Direct Effect
CI: Confidence Interval
GCSE: General Certificate of Secondary Education
NS-SEC: National Statistics Socioeconomic Classification
OR: Odds Ratio
SEP: Socioeconomic Position
UK: United Kingdom
